# Are There Differences between the Stress Responses of Philippine Men and Women to the COVID-19 Pandemic?

**DOI:** 10.3390/ijerph20032326

**Published:** 2023-01-28

**Authors:** Divya Periyakoil, Preethi Periyakoil, Cherica A. Tee, Costas J. Spanos, Marie Diener-West, Michael Tee, Ndola Prata

**Affiliations:** 1 Departments of Biostatistics and Epidemiology, Johns Hopkins Bloomberg School of Public Health, Baltimore, MD 21205, USA; 2 School of Public Health, University of California, Berkeley, CA 94720, USA; 3 Department of Electrical Engineering and Computer Science, University of California, Berkeley, CA 94720, USA; 4 Weill Cornell Medical College, Cornell University, New York, NY 10065, USA; 5 California Institute of Technology, Pasadena, CA 91125, USA; 6 College of Medicine, University of the Philippines Manila, Manila 1000, Philippines

**Keywords:** women’s health, mental health, health disparities during the COVID-19 pandemic

## Abstract

The SARS-CoV-2 pandemic has had a deleterious impact on human health since its beginning in 2019. The purpose of this study was to examine the psychosocial impact of the COVID-19 pandemic in the Philippines and determine if there were differential impacts on women compared to men. A web-based survey was conducted in the Luzon Islands of the Philippines, during the pandemic quarantine. A total of 1879 participants completed online surveys between 28 March–12 April 2020. A bivariate analysis of both men and women for each psychological measure (stress, anxiety, depression, and impact of COVID-19) was conducted. Multivariable logistic regression models were built for each measure, dichotomized as high or low, separately for men and women. Younger age (*p* < 0.001), being married (*p* < 0.001), and being a parent (*p* < 0.004) were associated with women’s poor mental health. Marriage and large household size are protective factors for men (*p* < 0.002 and *p* < 0.0012, respectively), but marriage may be a risk factor for women (*p* < 0.001). Overall, women were disproportionately negatively impacted by the pandemic compared to men.

## 1. Introduction

The SARS-CoV-2 pandemic has had a deleterious impact on human health since its beginning in 2019. As of 13 November 2022, over 630 million cases of COVID-19 have been confirmed, with more than 6.58 million deaths globally [1]. According to a meta-analysis published by Nochaiwong et al., the prevalence of mental illness has also increased globally since the start of the pandemic; the estimated global prevalence is 28.0% for depression, 26.9% for anxiety, 36.5% for stress, and 50.0% for psychological distress, all of which are significant increases from their prevalence pre-pandemic (which was estimated at 29.1% for all mental health disorders, 9.6% for mood disorders, and 12.9% for anxiety disorders) [2]. A scientific brief released by the World Health Organization found that the prevalence of depression and stress increased by nearly 25% in the first year, and as a direct result, of the COVID-19 pandemic [3]. The brief also found that young people and women were the most severely psychologically impacted by the COVID-19 pandemic.

While the mental health of the global population as a whole has been negatively affected by COVID-19, data show that women and girls have been disproportionately affected by mental health issues since the onset of the pandemic [4]. According to CARE’s Rapid Gender Analysis of mental illnesses across 38 countries, not only have women experienced a greater increase in depression and anxiety, but the number of women who reported mental health problems was three times higher than that of men. In addition to the labor increase faced by female frontline workers, domestic violence and post-traumatic stress disorder rates have also increased among women across the globe [4]. More women have reported utilizing shelters, hotlines, and other resources since the start of the pandemic, suggesting that their problems had increased in severity and that they required the use of outside resources to cope [4]. The International Journal of Mental Health has also reported that the mental health of women has been much more affected by the pandemic than that of men [5]. According to Lancet Global Health, mental health problems and the resulting decrease in productivity are estimated to cost the global economy USD 6 trillion by 2030, and the exacerbation of mental health issues caused by the pandemic are likely to substantially increase this cost [6].

The purpose of this study was to examine the psychosocial impact of the COVID-19 pandemic in the Philippines and to determine if there were differential impacts on women compared to men. A 2020 report by UN Women presented the “distinct gendered impacts” of the COVID-19 pandemic, specifically mentioning that the pandemic has been injurious to the agency and safety of women. According to the report, the pandemic has had deleterious effects on the mental and physical health of women in the Philippines. We wished to investigate the specific factors that either put women at risk for, or were protective of, mental health problems during the pandemic.

## 2. Data and Methods

### 2.1. Data Collection

This study analyzes data from the country of the Philippines collected as a part of a large multinational study entitled, “Psychological Responses and Associated Factors During the 2019 Coronavirus Diseases (COVID-19) Pandemic Among the General Population in Different Countries in Asia” [7]. As a result of the COVID-19 pandemic, the Luzon Islands of the Philippines were under an extended period of quarantine. During this time, a web-based survey was launched and disseminated through social media via the snowball sampling technique from 28 March–12 April 2020 to 2700 individuals. The survey, which consisted of 42 questions, was accessible through a secure server to assure privacy. Instead of names, subject numbers were used to ensure participant confidentiality. Further details about the creation and dissemination of the survey have been published previously [7]. A total of 2037 (75% response rate) individuals completed the survey. As the survey questions were set to require a response, only participants who completed all the questions were able to submit the survey. A total of 158 survey respondents, all of whom who had a history of neuropsychiatric conditions, were excluded from the study. This particular study was limited to participants without chronic mental health conditions, as the primary research question concerned the negative mental health impact of COVID. The remaining 1879 respondents served as the study sample representing the target population, which was the entire Philippines population. Collected data included socio-demographics, health status, contact history, COVID-19 knowledge and concerns, precautionary measures, information needs, the Depression, Anxiety and Stress Scales (DASS-21), and the Impact of Events Scale-Revised (IES-R) ratings. Socio-demographic variables that were collected in the survey and hypothesized to be potential factors associated with the psychosocial measures were used in the analysis. Such factors included the age of the respondent, marital status, household size, parental status, employment status, education, whether or not the respondent traveled, and if the respondent was a healthcare professional. Our study was approved by the Research Ethics Board of the University of the Philippines Manila (UPMREB 2020-198-01).

### 2.2. Measures

Four measures that collectively described the psychological impacts of COVID-19 were estimated based on survey response scales. For each measure, individual scores were used to create the following categories: “Not/Normal,” “Mild,” “Moderate,” and “Severe.” We measured the psychological impact of the COVID-19 pandemic using the Impact of Event Scale (IESR) and measured stress, anxiety, and depression using the Depression, Anxiety, and Stress Scale (DASS-21); a psychometrically robust self-reporting measure was utilized to assess their mental health. The DASS-21 has seven items that assess stress; participants read each statement and circle a number 0, 1, 2, or 3 indicating how much the statement applies to them. The total stress score range is from 0–21. We used the methodology of prior studies conducted using the same dataset to calculate each of the stress, anxiety, depression, and IESR scores [7,8]. Similar to th methods of prior studies, we used these scores to dichotomize each study participant as stressed/not stressed, depressed/not depressed, anxious/not anxious, and IESR/not IESR.

### 2.3. Statistical Analysis

All data analysis was performed using the statistical software Stata Version 16(5) [9]. A bivariate analysis for men and women for each psychological measure and the socio-demographic factors was conducted using the measure’s categories, and Chi-square tests and associated *p*-values were estimated. Variables associated with *p* < 0.2 at the bivariate level were selected for the multivariable models. Logistic regression models were estimated for each of the psychosocial measures in order to assess whether gender was significantly associated with each measure. Significance was established at *p* < 0.05. Multivariable logistic regression models were then built separately for each of the psychosocial measures for men and women.

## 3. Results

We conducted our analysis with a sample size of 1897 individuals. Calculations were performed to ensure that this sample size was representative of the population and more than adequate to answer the research questions under study. There was no missing data, so imputation was not needed. The demographics of the survey respondents are shown in Table 1. The sample was relatively equilibrated for men and women, with most of the respondents reporting an age of 35 years or older; most were never married; more than half were health care professionals living in households with 2–5 people; and the majority did not have parental duties. More than half of the respondents were employed at the time of the survey, and the vast majority had an educational level equal to or greater than a college degree, and they had also traveled.

**Figure 1:**
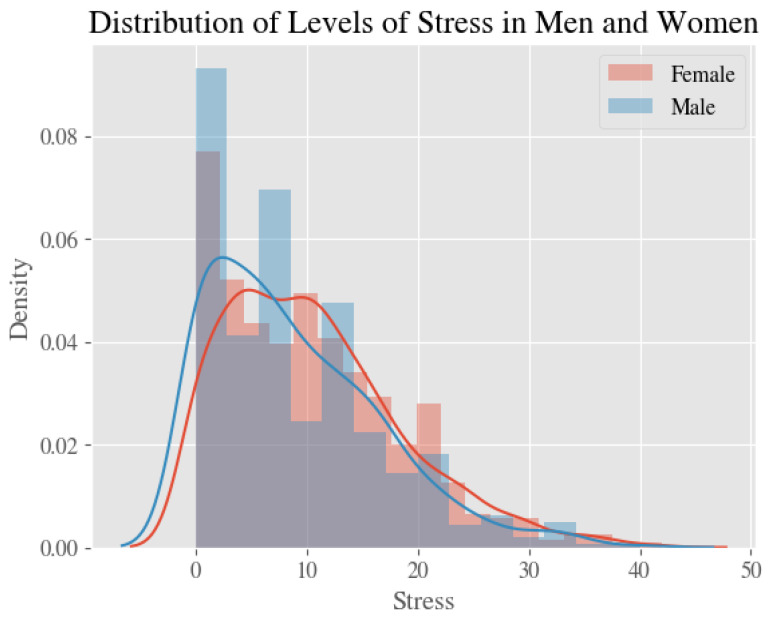
The Distribution of Levels of Stress in Men and Women.

**Figure 2:**
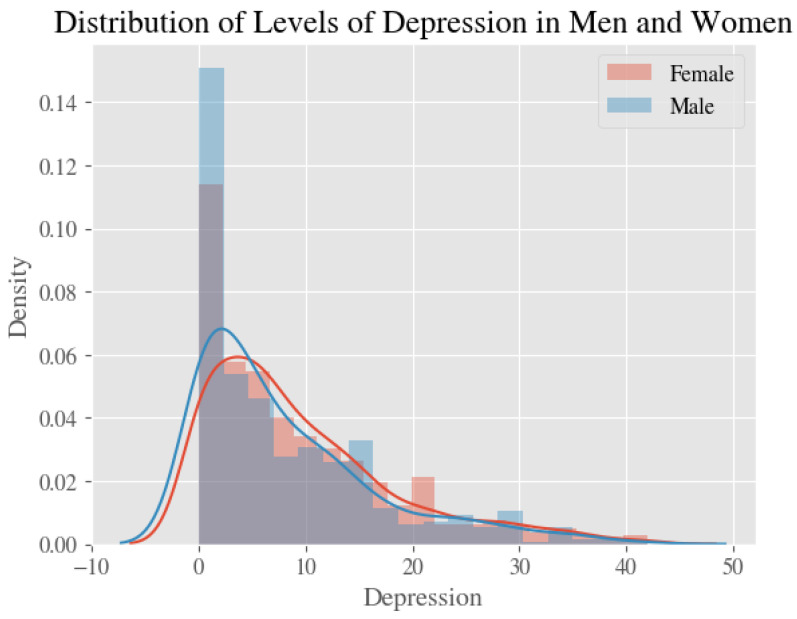
The Distribution of Levels of Depression in Men and Women.

**Figure 3:**
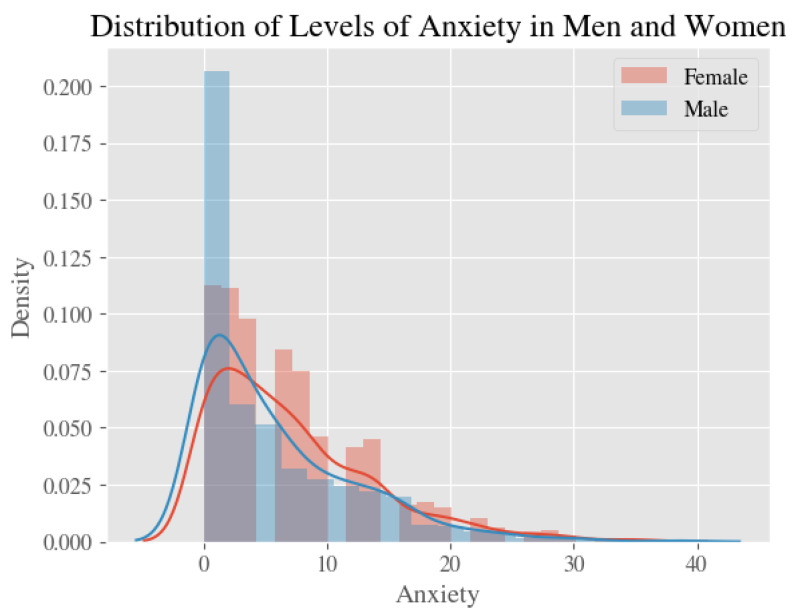
The Distribution of Levels of Anxiety in Men and Women.

**Figure 4:**
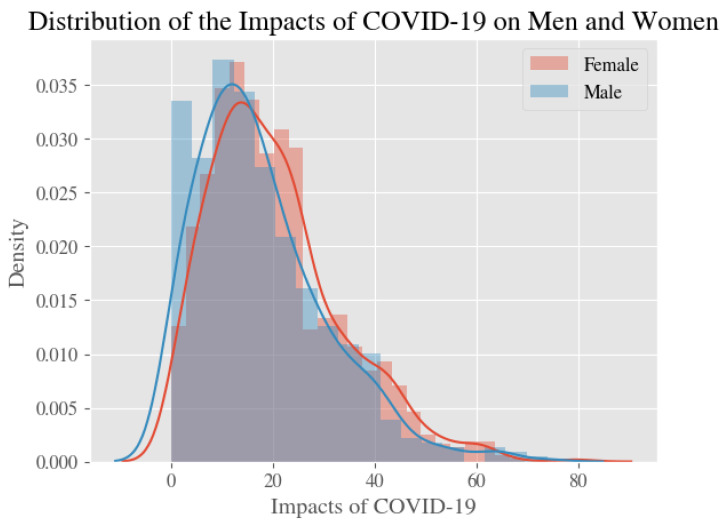
The Distribution of Levels of the Impacts of COVID-19 in Men and Women.

Average estimates and 95% confidence intervals for our four measures—stress, anxiety, depression, and the psychological impact of COVID—for all respondents, male respondents only, and female respondents only, are shown in Table 2. Figure 1, Figure 2, Figure 3 and Figure 4 show the distribution of the psychological measures by gender.

The results of the bivariate analysis for men and women are shown in Table 3a,b, respectively. We identified the socio-demographic factors that were significantly associated (*p* < 0.2) with psychosocial well-being among men and women at the bivariate level.

We found that for women, stress is significantly associated with age, marital status, parental status, employment status, and having traveled outside the Philippines within 14 days prior to data collection. Depression in women is significantly associated with age, marital status, and parental status, while anxiety is significantly associated with age, marital status, and parental status. The impacts of COVID-19 are significantly associated with age, marital status, healthcare professional status, and employment status.

We found that for men, stress is significantly associated with age, marital status, status as a healthcare professional, household size, parental status, employment status, and having traveled outside the Philippines within 14 days prior to data collection. Depression is significantly associated with age, marital status, healthcare professional status, household size, parental status, and employment status. Anxiety in men is significantly associated with age, marital status, healthcare professional status, household size, parental status, and employment status. The impacts of COVID-19 are significantly associated with age, marital status, healthcare professional status, parental status, employment status, and education status.

By building the unadjusted logistic regression model, we sought to understand how psychosocial well-being (dichotomized as high versus low) varies with respect to gender. The results of this model are presented in Table 4. The results showed that the extent to which men and women experience stress, depression, anxiety, and IESR is significantly different. We found that males are 49% less likely to be stressed, 39% less likely to be depressed, 44% less likely to have anxiety, and 54% less likely to feel the impacts of COVID-19 than their female counterparts. With the goal of understanding which socio-demographic factors contribute to their different experiences, for each of the psychosocial measures, we built two adjusted logistic regression models, one for each gender cohort.

### 3.1. Adjusted Model

We adjusted each multivariable logistic regression model by the features that were found to be significant (*p* < 0.20) at the bivariate level for every combination of psychosocial measure and gender cohort. The results with respect to stress, depression, anxiety, and the impacts of COVID-19 are presented in Table 5a–d, respectively.

### 3.2. Stress

For women, age was the main determinant of stress. Women 35 years of age or older were significantly less stressed than women younger than 35 years old. For men, none of the demographic factors that are significant at the bivariate level were significantly associated with stress effects.

### 3.3. Depression

For women, age continued to be the main determinant of depression. Women 35 years old or older were significantly less depressed than women younger than 35 years of age. For men, household size was the main determinant of depression. Men with a household size greater than one person were significantly less depressed than men who lived alone. Men who lived in a household of 2–5 people were 58% less depressed than men who lived alone.

### 3.4. Anxiety

For women, age was once again the main determinant of anxiety. Women 35 years old or older had significantly less anxiety than women younger than age 35. For men, none of the demographic factors that were significant at the bivariate level were significantly associated with the effects of anxiety.

### 3.5. Impacts of COVID-19

For women, age was again the main determinant of the impacts of COVID-19. Women 35 years old or older felt the impacts of COVID-19 to a significantly lesser degree than women younger than age 35. For men, marital status and healthcare professional status were the main determinants of the impacts of COVID-19. Men who were married were 63% less likely to feel the impacts of COVID-19 than men who were not married. Men who were healthcare professionals were 78% more likely to feel the impacts of COVID-19 than men who were not healthcare professionals.

## 4. Discussion

Overall, compared to men, our data showed that women on average reported significantly higher levels of stress, depression, and anxiety and were more adversely impacted by the pandemic. Studies conducted over the course of the COVID-19 pandemic have demonstrated a disproportionate burden that has been placed on women as a result of the pandemic [5]. Women have multiple responsibilities, including taking care of the household, childcare responsibilities, caregiving responsibilities of older members of the family, as well as professional roles and obligations [4]. UN Women, the United Nations’ gender equality agency, reported a 60% drop in income for women during the first month of the pandemic [10]. A study performed by the McKinsey & Company consulting firm reported that women face economic impacts of the pandemic to a much greater degree due to pre-existing inequalities and that women are almost twice as likely to lose their jobs than men during the pandemic [11]. Researchers from the London School of Economics reported that “macro factors”—such as the fact that women largely work in the industries most impacted by the pandemic (hospitality, tourism, restaurants, etc.)—and “micro factors”—such as the fact that some families have decided that the father should keep his job, while the mother should stay at home and support the children during the pandemic—have had a strong negative impact on the agency of women in the workforce as a result of the pandemic [4]. This lack of agency has a significant impact on their psychosocial well-being. Studies have shown that women are already more susceptible to stress, anxiety, and depression than their male counterparts, and experts posit that this is because women tend to hold lower-paying jobs and are depended upon as caregivers for their children and their families [4]. As of May 2020, the Centers for Disease Control and Prevention (CDC) reported that from the data they collected, 44% percent of women who answered their survey showed symptoms of anxiety or depression, as opposed to 36% of men who answered their survey [12].

Through our analysis, we found that age was consistently the main determinant of each of the four psychosocial factors for women. Younger women (<35 years of age) were more likely to have stress, depression, and anxiety or to feel the impacts of COVID-19. These results are supported by the fact that the median childbearing age among women in the Philippines is 27, which may imply that mothers of age 35 years or older are more experienced and resilient than newer mothers [13]. A Yahoo News poll reported that the pandemic has had a disproportionately negative effect on the mental health of mothers with children under the age of 18, while the mental health of fathers with children under the age of 18 is largely unchanged [14].

We found that household size is the main determinant of depression in men and that unmarried men were more likely to feel the impacts of the pandemic than men who were married. This implies that marriage is a protective factor for men, but may be a risk factor for women. A study conducted in Germany by Johannes Gutenberg University found that of the respondents to their survey who reported feelings of loneliness, 76% also reported symptoms of anxiety, and 78% reported symptoms of depression [15]. Another study conducted by National Taiwan University found that the social isolation brought about by the pandemic had a disproportionately negative impact on the mental health of those with pre-existing mental illnesses [16]. Finally, a study conducted in Pennsylvania by Cabrini University found that perceived social isolation was associated with stress and poor life satisfaction, especially among young adults [17]. A systematic review of papers assessing the psychological impacts of COVID-19 found that female gender was a “direct and independent risk factor for developing abnormal stress symptoms.” The review also concluded that being young and female was “significantly associated with more negative psychological impacts of COVID-19,” as well as “higher levels of stress, anxiety, and depression” [18]. Although prior research has concluded that females in the Philippines were more negatively psychologically affected by the COVID-19 pandemic than males, they do not explain the social, economic, or demographic factors that may have contributed that this outcome [7]. Our research is filling the current gaps in the existing research by studying the factors that affected the mental health of women during the pandemic in the Philippines. Additionally, as we have studied the social factors that affect mental health in the Philippines, these results can be applied to similar developing countries.

Our study also found that men who were healthcare professionals were more likely to feel the impacts of the pandemic. In a study conducted by the Norwegian Institute of Public Health, healthcare workers reported feeling anxiety and depression during the COVID-19 pandemic as a result of systematic factors such as an increase in workload and possible exposure to COVID-positive patients. The World Health Organization (WHO) also emphasized the disproportionate physical and psychological burdens being placed on healthcare workers as they respond to the urgent need for health care during the pandemic [19].

Our study has limitations. First, we analyzed the Philippine country data from a larger study involving multiple countries in Asia. Our institutional research board was based in the Philippines and provided permission to analyze this data. Work is ongoing to acquire and analyze data from other Asian countries which will allow us to make comparisons across the countries. It is also to be noted that some studies claim that women are more likely than men to report mental health symptoms [20,21]. Moreover, this is a web-based study, and therefore, it excludes participants who do not have internet access. However, participants were able to access the study portal through their cell phones, and most people in Luzon Island have cell phones. This study is a cross-sectional study referring to the early part of the pandemic. The pandemic is still raging across the globe, and data collection is still ongoing: we will analyze the data to assess longitudinal patterns once the large multi-national study is complete. While the questions in the survey are used to gauge levels of three psychosocial measures specifically in the context of the pandemic, it is possible that the participants had other sources of stress, anxiety, and depression that were captured by the measures. Conversely, the strength of this study is the existence of a separate measure for the impact of COVID-19, which shows the same trends as the other three measures with respect to gender. The impact of the COVID-19 measures cannot be confounded with any pre-existing conditions of stress, anxiety, or depression, because these impacts are specific with respect to the pandemic.

### Public Health Implications

A great deal of recent research studying the effects of the pandemic on psychosocial factors and mental well-being has been gender neutral. Our work provides a scientific basis for understanding how demographic and socioeconomic factors differentially affected, and continue to affect, the psychosocial well-being of men and women during the COVID-19 pandemic. Research from the Global Public Health Department of University College London has stated that governments must assume the responsibility “to plan and implement interventions that are gender-responsive” [4]. As an example, provisions must be made to protect women and children who are at risk of domestic violence and sexual assault within their own homes” [4]. The study went on to say that the pandemic has caused social equality to regress, and that gender equality and the well-being of women has been seen as an afterthought, paling in “importance” to the more universal effects of the pandemic. Our study has patently demonstrated a disproportionate disparity between the mental health of men and women, with women being affected even more severely in the wake of the pandemic [4]. The United Nations’ 2030 Agenda for Sustainable Development states the importance of “realizing gender equality and the empowerment of women and girls,” claiming that “women and girls must enjoy equal access to quality education, economic resources, and political participation, as well as equal opportunities with men and boys for employment, leadership, and decision-making at all levels” [22]. However, these goals cannot be realized without gaining a deeper understanding of the differential treatment of men and women, and how this affects women’s mental health. The pandemic has only further widened this disparity, and it is imperative that we first acknowledge and then address the problem.

## 5. Conclusions

This study showed that for all four measures, stress, anxiety, depression, and IESR, on average, women reported significantly higher levels of psychological distress than men, demonstrating that the mental health of men and women has been differently impacted during the pandemic in the Philippines. Moreover, socio-demographic factors appear to impact how stress is experienced in both men and women. Our research has identified some of the social gaps that have to be addressed as part of an overall effort to eliminate gender disparities. The insights gained from this analysis are essential inputs in gender mainstreaming. Specifically, it can guide actions in ensuring better design, implementation, monitoring, and evaluation of policies, programs, and projects in the national efforts to create to a new post-pandemic normal [23].

## Figures and Tables

**Table 1:** Participant demographics.

Characteristics	Total N = 1879 (100%)	Men N = 583 (31%)	Women N = 1296 (69%)
Age			
≤19	11.50%	11.70%	11.40%
20–24	20.50%	17.30%	22.00%
25–29	10.60%	11.00%	10.50%
30–34	11.70%	12.50%	11.30%
≥35	45.60%	47.50%	44.80%
Marital Status			
Never Married	63.70%	65.20%	63.00%
Ever Married	36.40%	34.80%	37.00%
Healthcare Professional			
No	33.40%	34.20%	33.00%
Yes	66.60%	65.80%	67.00%
Household Size			
1 person	5.70%	5.80%	5.60%
2–5 people	63.60%	66.70%	62.20%
≥6 people	30.70%	27.40%	32.20%
Parental Status			
No	65.80%	67.90%	64.80%
Yes	34.20%	32.10%	35.20%
Employment Status			
Unemployed	38.40%	36.70%	39.20%
Employed	61.60%	63.30%	60.80%
Education Status			
<College	7.08%	7.03%	7.10%
≥College	92.90%	93.00%	92.90%
Traveled			
No	1.70%	1.50%	1.70%
Yes	98.40%	98.50%	98.30%

**Table 2:** Psychosocial well-being by gender.

Measures	Total (Mean, 95% CI)	Among Men (Mean, 95% CI)	Among Women (Mean, 95% CI)
Stress [0–42]	10.1 (9.75–10.47)	8.9 (8.29–9.55)	10.6 (10.21–11.08)
Anxiety [0–32]	6.8 (6.50–7.09)	6.0 (5.45–6.49)	7.2 (6.80–7.52)
Depression [0–42]	9.0 (8.64–9.43)	8.1 (7.44–8.85)	9.4 (8.96–9.91)
IESR [0–81]	19.6 (19.0–20.2)	17.7 (16.66–18.75)	20.4 (19.69–21.12)

**Table 3:** Bivariate analysis of socio-demographic factors and psychosocial well-being among men and women.

**a**: Bivariate analysis of socio-demographic factors and psychosocial well-being among men.				
**Socio-Demographic Factors**	**% Not Stressed**	**% Not Depressed**	**% Not Anxious**	**% Not Impacted by COVID (IESR Scale)**
Age				
≤19	11.60%	11.30%	11.50%	10.80%
20–24	13.10%	14.30%	13.50%	15.70%
25–29	10.50%	10.80%	10.40%	10.00%
30–34	13.10%	11.30%	11.20%	11.50%
≥35	51.70%	52.30%	53.40%	52.10%
	(*p =* 0.019)	(*p =* 0.000)	(*p =* 0.001)	(*p =* 0.009)
Marital Status				
Never Married	59.80%	60.40%	58.50%	60.80%
Ever Married	40.20%	39.60%	41.50%	39.20%
	(*p =* 0.000)	(*p =* 0.000)	(*p =* 0.000)	(*p =* 0.002)
Healthcare Professional				
No	35.60%	35.00%	36.70%	38.10%
Yes	64.40%	65.00%	63.30%	61.90%
	(*p =* 0.115)	(*p =* 0.048)	(*p =* 0.001)	(*p =* 0.004)
Household Size				
1 person	6.00%	4.38%	4.30%	5.10%
2–5 people	66.40%	68.00%	67.20%	67.80%
≥6 people	27.60%	27.70%	28.50%	27.10%
	(*p =* 0.001)	(*p =* 0.001)	(*p =* 0.012)	(*p =* 0.777)
Parental Status				
No	63.50%	64.30%	62.60%	65.20%
Yes	36.50%	35.70%	37.40%	34.80%
	(*p =* 0.004)	(*p =* 0.002)	(*p =* 0.000)	(*p =* 0.034)
Employment Status				
Unemployed	34.10%	35.00%	34.90%	34.10%
Employed	65.90%	65.00%	65.10%	65.90%
	(*p =* 0.032)	(*p =* 0.103)	(*p =* 0.160)	(*p =* 0.011)
Education Status				
<College	6.80%	7.40%	7.10%	6.80%
≥College	93.20%	92.60%	92.90%	93.20%
	(*p =* 0.764)	(*p =* 0.508)	(*p =* 0.304)	(*p =* 0.009)
Traveled				
No	1.80%	1.60%	1.30%	1.60%
Yes	98.20%	98.40%	98.70%	98.40%
	(*p =* 0.182)	(*p =* 0.353)	(*p =* 0.326)	(*p =* 0.919)
**b**: Bivariate analysis of socio-demographic factors and psychosocial well-being among women.				
**Socio-demographic factors**	**% Not Stressed**	**% Not Depressed**	**% Not** **Anxious**	**% Not Impacted by COVID** **(IESR Scale)**
Age				
≤19	11.10%	9.70%	9.80%	10.00%
20–24	17.90%	20.10%	19.60%	19.40%
25–29	11.10%	10.00%	9.70%	9.00%
30–34	10.80%	11.30%	11.10%	11.00%
≥35	49.20%	48.90%	49.70%	50.50%
	(*p* = 0.000)	(*p* = 0.000)	(*p* = 0.000)	(*p* = 0.000)
**Marital Status**				
Never Married	59.50%	59.50%	59.00%	59.60%
Ever Married	40.50%	40.50%	41.00%	40.40%
	(*p* = 0.000)	(*p* = 0.001)	(*p* = 0.001)	(*p* = 0.001)
**Healthcare Professional**				
No				
Yes	33.70%	34.40%	35.70%	35.80%
	66.30%	65.60%	64.30%	64.30%
	(*p* = 0.458)	(*p* = 0.233)	(*p* = 0.201)	(*p* = 0.015)
**Household Size**				
1 person	5.70%	5.60%	5.80%	5.40%
2–5 people	63.70%	62.00%	62.20%	62.30%
≥6 people	30.50%	32.40%	32.10%	32.30%
	(*p* = 0.619)	(*p* = 0.790)	(*p* = 0.975)	(*p* = 0.686)
**Parental Status**				
No	61.70%	61.60%	61.10%	61.70%
Yes	38.30%	38.40%	38.90%	38.30%
	(*p* = 0.000)	(*p* = 0.004)	(*p* = 0.002)	(*p* = 0.002)
**Employment Status**				
Unemployed	38.30%	37.80%	38.20%	37.10%
Employed	61.70%	62.20%	61.80%	62.90%
	(*p* = 0.053)	(*p* = 0.491)	(*p* = 0.244)	(*p* = 0.084)
**Education Status**				
<College	7.50%	6.30%	6.70%	7.30%
≥College	92.50%	93.70%	93.30%	92.70%
	(*p* = 0.603)	(*p* = 0.531)	(*p* = 0.252)	(*p* = 0.263)
**Traveled**				
No	1.70%	1.60%	1.40%	1.20%
Yes	98.30%	98.50%	98.60%	98.80%
	(*p* = 0.166)	(*p* = 0.710)	(*p* = 0.214)	(*p* = 0.224)

**Table 4:** The extent to which men and women experience stress, depression, anxiety, and IESR is significantly different (unadjusted model).

	Stress OR, CI, *p*-Value *	Depression OR, CI, *p*-Value *	Anxiety OR, CI, *p*-Value *	IESR OR, CI, *p*-Value *
Women (ref)	1	1	1	1
Men	0.51 (0.38, 0.68) ***	0.70(0.54, 0.90) **	0.56(0.44, 0.71) ***	0.46 (0.25, 0.86) *

Notes: * *p* < 0.05; ** *p* < 0.01; *** *p* < 0.001.

**Table 5:** Factors associated with psychosocial well-being among men and women (adjusted model).

	Men Odds Ratio, CI, *p*-Value *	Women Odds Ratio, CI, *p*-Value *
(a) Stress		
Age		
≤19		
20–24	1.77 (0.95, 3.36)	1.33 (0.89, 2.00)
25–29	0.98 (0.41, 2.32)	0.63 (0.36, 1.08)
30–34	0.87 (0.36, 2.11)	0.80 (0.46, 1.39)
≥35	0.89 (0.39, 2.06)	0.58 (0.34, 0.98) *
Marital Status		
Never Married		
Ever Married	0.61 (0.30, 1.25)	0.94 (0.62, 1.41)
Healthcare Professional		
No		
Yes	0.95 (0.62, 1.44)	
Household Size		
1 person		
2–5 people	1.22 (0.54, 2.76)	
≥6 people	1.10 (0.46, 2.62)	
Parental Status		
No		
Yes	0.90 (0.45, 1.81)	1.02 (0.69, 1.51)
Employment Status		
Unemployed		
Employed	1.21 (0.70, 2.10)	1.37 (1.00, 1.88)
Education Status		
<College		
≥College		
Traveled		
No		
Yes	1.79 (0.35, 9.04)	0.90 (0.38, 2.15)
(b) Depression:		
	**Men** Odds Ratio, CI, *p*-Value *	**Women** Odds Ratio, CI, *p*-Value *
Age		
≤19		
20–24	1.38 (0.70, 2.72)	0.83 (0.55, 1.25)
25–29	0.62 (0.24, 1.62)	0.76 (0.47, 1.23)
30–34	0.91(0.35, 2.34)	0.68 (0.42, 1.11)
≥35	0.50 (0.20, 1.25)	0.52 (0.32, 0.82) *
Marital Status		
Never Married		
Ever Married	0.46 (0.20, 1.04)	0.91 (0.58, 1.42)
Healthcare Professional		
No		
Yes	0.86 (0.54, 1.37)	
Household Size		
1 person		
2–5 people	0.42 (0.19, 0.94) *	
≥6 people	0.39 (0.16, 0.92) *	
Parental Status		
No		
Yes	1.39 (0.63, 3.06)	0.93 (0.60, 1.42)
Employment Status		
Unemployed		
Employed		
	1.47 (0.79, 2.71)	
Education Status		
<College		
≥College		
Traveled		
No		
Yes		
(c) Anxiety		
	**Men** Odds Ratio, CI, *p*-Value *	**Women** Odds Ratio, CI, *p*-Value *
Age		
≤19		
20–24	1.58 (0.83, 3.03)	0.92 (0.62, 1.38)
25–29	0.80 (0.33, 1.96)	0.87 (0.54, 1.38)
30–34	1.14 (0.47, 2.76)	0.77 (0.48, 1.24)
≥35	0.68 (0.29, 1.60)	0.58 (0.37, 0.91) *
Marital Status		
Never Married		
Ever Married	0.46 (0.22, 0.97)	0.95 (0.63, 1.43)
Healthcare Professional		
No		
Yes	1.12 (0.73, 1.73)	
Household Size		
1 person		
2–5 people	0.50 (0.23, 1.11)	
≥6 people	0.43 (0.18, 1.00)	
Parental Status		
No		
Yes	1.16 (0.56, 2.41)	0.94 (0.64, 1.39)
Employment Status		
Unemployed		
Employed	1.44 (0.82, 2.56)	
Education Status		
<College		
≥College		
Traveled		
No		
Yes		
(d) IESR		
	**Men** Odds Ratio, CI, *p*-Value *	**Women** Odds ratio, CI, *p*-Value *
Age		
≤19		
20–24	1.11 (0.56, 2.23)	0.98 (0.65, 1.48)
25–29	1.43(0.55, 3.76)	0.95 (0.55, 1.65)
30–34	1.79 (0.69, 4.66)	0.66 (0.37, 1.18)
≥35	1.05 (0.41, 2.64)	0.36 (0.21, 0.64) ***
Marital Status		
Never Married		
Ever Married	0.37 (0.16, 0.82) *	1.22 (0.78, 1.91)
Healthcare Professional		
No		
Yes	1.78 (1.10, 2.86) *	1.22 (0.92, 1.60)
Household Size		
1 person		
2–5 people		
≥6 people		
Parental Status		
No		
Yes	1.68 (0.77, 3.63)	1.08 (0.71, 1.65)
Employment Status		
Unemployed		
Employed	0.95 (0.53, 1.70)	1.17 (0.84, 1.63)
Education Status		
<College		
≥College	0.95 (0.62, 1.42)	
Traveled		
No		
Yes		

Notes: * *p* < 0.05; *** *p* < 0.001; for men adjusted for XXXXXX-; women adjusted for xxxxxx.

## Data Availability

Restrictions apply to the availability of these data. Data was obtained from the University of the Philippines, Manila and are available at the discretion of the data curators.
